# Automated evaluation of masseter muscle volume: deep learning prognostic approach in oral cancer

**DOI:** 10.1186/s12885-024-11873-y

**Published:** 2024-01-24

**Authors:** Katsuya Sakamoto, Shin-ichiro Hiraoka, Kohei Kawamura, Peiying Ruan, Shuji Uchida, Ryo Akiyama, Chonho Lee, Kazuki Ide, Susumu Tanaka

**Affiliations:** 1 Department of Oral and Maxillofacial Surgery, Graduate School of Dentistry, Osaka University, 1-8 Yamada-Oka, 565-0871 Suita, Osaka Japan; 2 NVIDIA AI Technology Center, NVIDIA Japan, 12F ATT New Tower, 2-11-7, Akasaka, Minato-ku, 107-0052 Tokyo, Japan; 3 Cybermedia Center, Osaka University, 5-1 Mihogaoka, 567-0047 Ibaraki city, Osaka Japan; 4 Division of Scientific Information and Public Policy, Center for Infectious Disease Education and Research, Research Center on Ethical, Legal and Social Issues, Osaka University, Osaka University, Techno-Alliance Building C 208, 2-8 Yamadaoka, 565-0871 Suita, Osaka Japan

**Keywords:** Oral cancer, Sarcopenia, Deep learning

## Abstract

**Background:**

Sarcopenia has been identified as a potential negative prognostic factor in cancer patients. In this study, our objective was to investigate the relationship between the assessment method for sarcopenia using the masseter muscle volume measured on computed tomography (CT) images and the life expectancy of patients with oral cancer. We also developed a learning model using deep learning to automatically extract the masseter muscle volume and investigated its association with the life expectancy of oral cancer patients.

**Methods:**

To develop the learning model for masseter muscle volume, we used manually extracted data from CT images of 277 patients. We established the association between manually extracted masseter muscle volume and the life expectancy of oral cancer patients. Additionally, we compared the correlation between the groups of manual and automatic extraction in the masseter muscle volume learning model.

**Results:**

Our findings revealed a significant association between manually extracted masseter muscle volume on CT images and the life expectancy of patients with oral cancer. Notably, the manual and automatic extraction groups in the masseter muscle volume learning model showed a high correlation. Furthermore, the masseter muscle volume automatically extracted using the developed learning model exhibited a strong association with life expectancy.

**Conclusions:**

The sarcopenia assessment method is useful for predicting the life expectancy of patients with oral cancer. In the future, it is crucial to validate and analyze various factors within the oral surgery field, extending beyond cancer patients.

## Background

Despite the advancements in the treatment that have improved the survival rates, oral cancer has the highest mortality rate among all types of head and neck cancers [1]. A total of 377,713 new cases and 177,757 deaths due to oral cancer were reported in 2020, making it the 18th most commonly diagnosed cancer worldwide [2]. In Japan, the number of oral cancer cases is increasing as the population ages, accounting for approximately 40% of all head and neck cancer cases. The male-to-female ratio is 3:2, with men outnumbering women; the majority of patients with oral cancer are in their 60s [3]. Recently, sarcopenia, characterized by the loss of muscle strength and mass, may be a poor prognostic factor in patients with cancer [4]. Patients with head and neck cancer and upper gastrointestinal cancer have a significantly higher risk of developing sarcopenia compared with patients with other cancer types, owing to severe nutritional disorders [5]. Patients with oral cancer may already have sarcopenia prior to the diagnosis of cancer due to undernutrition and weight loss caused by difficulty with oral intake. However, only a few studies have reported the occurrence of sarcopenia in patients diagnosed with oral cancer; therefore, the actual incidence of sarcopenia remains unclear.

The Asian Working Group for Sarcopenia (AWGS) [6] diagnostic criteria include the skeletal muscle index (SMI) determined by bioelectrical impedance analysis (BIA) to assess the limb skeletal muscle mass. Recently, a sarcopenia assessment method using the area [7] and volume [8] of the psoas major muscle measured at the level of the third lumbar vertebra (L3) on computed tomography (CT) images in patients with gastroin-testinal cancer has been reported and is associated with life expectancy. However, assessing the psoas major muscle at the L3 level is difficult as abdominal CT imaging is not routinely performed in patients with oral cancer. Wallace et al. [9] reported that the cross-sectional area of the masseter muscle correlates with the L3-level psoas major cross-sectional area on CT images in older patients with trauma. Yoshimura et al. re-ported that cervical (C3) skeletal muscle mass measured on CT may be associated with the favorable prognosis in patients with oral squamous cell carcinoma [10](1). The masseter muscle plays an important role in performing masticatory movements. The masseter muscle cross-sectional area directly correlates with bite force [11], and the maximum bite force is associated with mortality [12].

In recent years, artificial intelligence (AI) has been rapidly implemented in society with advancements in hardware, such as graphic processing units, faster Internet speeds, and the widespread use of cloud storage. Using deep learning, the methods used for automatically detecting polyps on colonoscopy images [13], the methods for classifying lung cancer using cytological diagnosis images [14], and research and development applying deep learning technology in the medical field are rapidly advancing.

This study aimed to investigate the association between a decrease in masseter muscle volume (MMV) on CT images and life expectancy in patients with oral cancer. In addition, to eliminate the bias caused by the evaluator’s manual extraction of the MMV data, we developed a learning model that automatically extracts the MMV data using deep learning and examined its clinical usefulness.

## Methods

### Patients

We included 348 patients (177 men and 171 women) admitted in the Department of Oral Surgery, Osaka University Dental Hospital (our department) and scheduled for surgery under general anesthesia between January 2017 and December 2020 (Table 1).

**Table 1:** Patient data used for setting the cutoff value

Characteristics	*n* = 348	
	N	Ratio (%)
Sex (male/female)	177/171	50.9/49.1
Age (years)		
Mean ± SD	41.0 ± 20.2	
Median (range)	33 (20–89)	
Case		
Jaw deformity	181	52
Malignant tumor	79	22.7
Cyst	35	10.1
Cleft lip and palate	16	4.6
Benign tumor	15	4.3
Fracture	11	3.1
Leucoplakia	3	0.9
Sialolith	3	0.9
Others^a^	5	1.4
CRP (mg/dL)	0.24 ± 0.47	
Alb (g/dL)	4.50 ± 0.36	
A/G	1.63 ± 0.28	
T-cho (mg/dL)	190.87 ± 32.14	
BMI (kg/m^2^)	21.79 ± 3.60	
CAR	0.06 ± 0.12	
NLR	2.29 ± 1.28	
PLR	155.13 ± 69.47	
PNI	53.92 ± 4.87	
mGPS (0/1/2)	331/14/3	95.1/4/0.9
CONUT (0/1/2/3/4/6)	95/145/77/24/6/1	27.3/41.7/22.1/6.9/1.7/0.3
SMI (kg/m^2^)		
Male	7.62 ± 0.85	
Female	5.88 ± 0.72	
MMV (cm^3^)		
Male	50.46 ± 12.43	
Female	32.16 ± 8.61	

aOthers: impacted wisdom tooth (*n* = 1), temporomandibular joint ankylosis (*n* = 1), unreduced dislocation of the temporomandibular joint (*n* = 1), residual fistula (*n* = 1), and foreign body on the oral floor (*n* = 1)

We used the data of these patient groups to determine the cutoff values. Head and neck CT and Body composition analysis by BIA were performed on all patients prior to treatment. We excluded patients aged < 20 years, those with infections that might affect nutrition-related factors, and those with a history of syndromes involving head and neck dysplasia.

We included 308 patients (176 men and 132 women) with oral cancer who received primary treatment at our department between January 2006 and December 2020 (Table 2).

**Table 2:** Patient data for validation

Characteristics	*n* = 308	
	N	Ratio (%)
Sex (male/female)	176/132	57.1/42.9
Age (years)		
Mean ± SD	62.7 ± 15.7	
Median (range)	66 (20–90)	
Tumor location		
Tongue/gingival/oral floor/buccal area	129/87/33/23	41.9/28.2/10.7/7.5
Palate/intraosseous^a^/maxillary sinus	16/8/5	5.2/2.6/1.6
Lip/submandibular gland/sublingual gland	3/2/2	1.1/0.6/0.6
Stage^b^		
I/II/III/IV	108/86/19/95	35.1/27.9/6.2/30.8
Treatment		
Operation (Ope)	222	72.1
Chemotherapy (Chemo)	2	0.6
Radiation therapy (RT)	2	0.6
Ope + Chemo/RT	68	22.1
Chemo + RT	14	4.6
CRP (mg/dL)	0.42 ± 0.60	
Alb (g/dL)	4.30 ± 0.44	
A/G	1.49 ± 0.31	
T-cho (mg/dL)	198.55 ± 37.01	
BMI (kg/m^2^)	21.60 ± 3.50	
CAR	0.10 ± 0.17	
NLR	2.52 ± 1.35	
PLR	156.12 ± 69.29	
PNI	51.50 ± 5.63	
mGPS (0/1/2)	264/38/6	85.7/12.3/2.0
CONUT (0/1/2/3/4/6/7)	101/100/59/33/12/2/1	32.8/32.5/19.2/10.7/3.9/0.6/0.3
MMV (cm^3^)		
Male	53.44 ± 14.50	
Female	36.21 ± 9.29	

aTreated intraosseous carcinoma as a gingival carcinoma

bAccording to the Union for International Cancer Control tumor-node-metastasis classification, 8th edition

The data of these patient groups were used for validation. CT imaging of the head and neck was performed in all patients prior to treatment. Exclusion criteria included patients with direct tumor invasion of the masseter muscle, previous surgical or nonsurgical treatment for oral cancer, and patients younger than 20 years of age who were still growing, in order to ensure that the masseter volume measurements were not directly influenced by the presence of a tumor.

This study was approved by the Ethical Review Committee of Osaka University Graduate School of Dentistry and Dental Hospital (approval no. H29-E19).

### Methods of measuring skeletal muscle index and masseter muscle volume

SMI was measured at the time of admission using the BIA method with InBody 570TM (InBody Japan). MMV was measured on the head and neck CT images obtained within 6 months prior to the initiation of treatment. Contrast or non-contrast CT imaging was performed using the following parameters: 2.5–5.0-mm slice thickness, 120 kVp, and 200–330 mA. The images acquired were converted from DICOM format to NIFTY format. Volume values were calculated by manually extracting both sides of the masseter muscle from the head and neck CT images using a three-dimensional slicer (version 4.11.0, www.slicer.org). The accuracy of the manual extraction of the masseter muscle was con-firmed by a specialist in our department (certified by the Japanese Society for Oral and Maxillofacial Radiology).

### Analysis factors

We collected the data on age, sex, stage (Union for International Cancer Control 8th edition [15]), and nutrition-related factors as analytic factors. Meanwhile, the following nutrition-related factors were obtained: C-reactive protein (CRP) (mg/dL), albumin (Alb) (g/dL), Alb/globulin (A/G) ratio, total cholesterol (T-cho) (mg/dL), body mass index (BMI) (kg/m2), CRP/Alb ratio (CAR) [16], neutrophil/lymphocyte ratio (NLR) [17], plate-let/lymphocyte ratio (PLR) [18], prognostic nutrition index (PNI) [19], modified Glasgow prognostic score (mGPS) [20], and controlling nutritional status (CONUT) score [21, 22]. CAR, NLR, PLR, and PNI were calculated as follows: CAR = CRP (mg/dL)/Alb (g/dL), NLR = neutrophil count/lymphocyte count, PLR = platelet count/lymphocyte count, and PNI = 10 × Alb (g/dL) + 0.005 × lymphocyte count. The mGPS was scored by combining CRP (mg/dL) and Alb (g/dL), while CONUT was scored by combining Alb (g/dL), total lymphocyte count, and T-cho (mg/dL). CAR, NLR, PLR, PNI, mGPS, and CONUT score were reported as prognostic predictors in patients with gastrointestinal cancer [16–22]. Among the data used for setting the cutoff values (Table 1), the cutoff values for age- and nutrition-related factors were set using the cutoff values for SMI in accordance with the AWGS [6] diagnostic criteria and using the receiver operating characteristic (ROC) curve.

### Development of the masseter muscle volume learning model

**Fig. 1:**
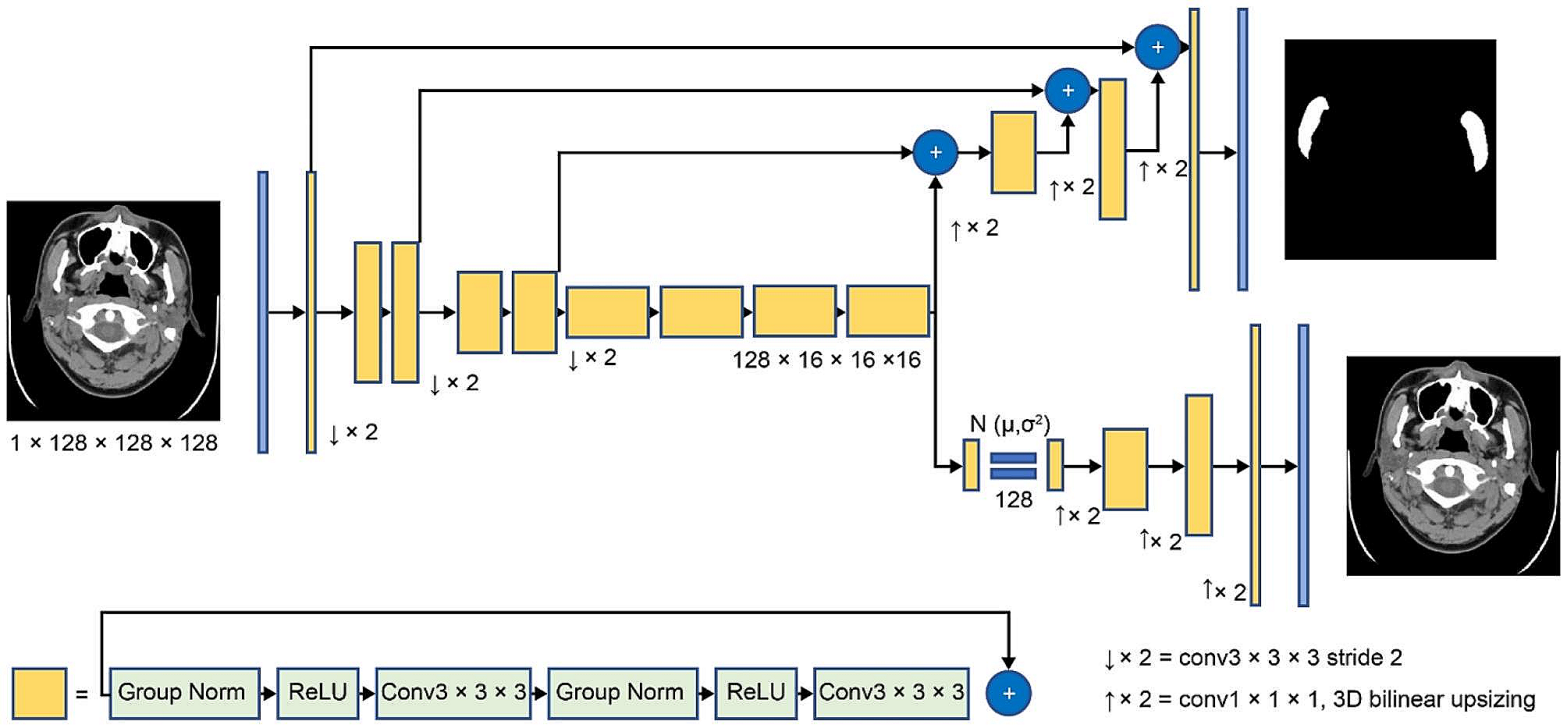
Schematic representation of SegResNet Adapted and partially modified from Andriy M. Springer: 311–320, 2018. Schematic overview of the deep learning architecture. The encoder part consists of normalization by group, a rectified linear unit, and 3 × 3 × 3 convolution, and the initial number of filters is 16. The decoder part consists of an upsizing and 1 × 1 × 1 convolution. The segmentation map is output with the same spatial size as the input image, and the input image is reconstructed. The input images are compressed to 128 × 128 × 128 voxel and are used as the network input

Clara Train SDK from NVIDIA Clara Imaging (Clara) (https://developer.nvidia.com/clara-medical-imaging) was used to develop the MMV learning model. Clara is a platform for medical imaging that has AI capabilities, such as medical image reconstruction, annotation, and segmentation using deep learning. Clara Train SDK is a Python-based application. Models can be trained by AI-assisted annotation of NVIDIA’s pretrained models, transfer learning using the individual institution’s own data, and automatic machine learning. In the 277 patients, after excluding the data of patients with oral cancer from those used for setting cutoff values (Table 1), the MMV manual extraction data and original CT image data were used as training data. We developed the MMV learning model by transferring a pretrained spleen volume model (https://catalog.ngc.nvidia.com/orgs/nvidia/teams/med/models/clara_pt_spleen_ct_annotation) based on SegResNet [23], which is included in the Clara Train SDK (Fig. 1).

In the validation data (Table 2), artificial intelligence masseter muscle volume (AIMMV) was defined as the automatically extracted masseter muscle volume.

### Items for investigation

#### Setting of cutoff values for masseter muscle volume

The MMV cutoff values for men and women were set using the cutoff values for SMI according to the AWGS [6] diagnostic criteria (men, < 7.0 kg/m2; women, < 5.7 kg/m2) and using the ROC curve. The patient data for setting cutoff values (Table 1) were used to set the cutoff values.

#### Evaluation of manually extracted masseter muscle volume

We evaluated the correlation between MMV and SMI using Pearson’s correlation coefficient. In the validation data (Table 2), the overall survival (OS) of the low MMV group was evaluated using the log-rank test. Moreover, a univariate analysis of the OS was performed using Fisher’s exact test after adjusting for age, sex, stage, nutrition-related factors, and low MMV, while a multivariate analysis of the factors that were significantly different was performed using the Cox proportional hazards regression model.

#### Evaluation of masseter muscle volume automatically extracted by the masseter muscle volume learning model

In the validation data (Table 2), we evaluated the correlation between manually extracted MMV and automatically extracted AIMMV using Pearson’s correlation coefficient. Then, we evaluated the OS of the low AIMMV group using the log-rank test. In addition, a univariate analysis of the OS was performed using Fisher’s exact test after adjusting for age, sex, stage, nutrition-related factors, and low AIMMV, while a multivariate analysis of the factors that were significantly different was performed using the Cox proportional hazards regression model.

### Statistical analyses

All statistical analyses were performed using EZR version 1.40, with a statistical significance level set at a *p*-value of < 0.05. Descriptive statistics for normally distributed continuous variables were presented as mean and standard deviation. Normality was investigated using the Kolmogorov–Smirnov test. Categorical variables were expressed as frequency (n) and ratio (%).

OS was measured from the date of primary treatment initiation to the date of death or final follow-up. Pearson’s correlation coefficient was used to analyze the correlation between MMV and SMI and between the manually extracted MMV and AIMMV auto-matically extracted by the MMV learning model. For comparative analysis of OS in the low and normal muscle mass groups, the log-rank test was used and visualized using Kaplan–Meier curves.

Fisher’s exact test and the Cox proportional hazards regression model were used for the univariate and multivariate analyses of OS after the adjusting for age, sex, stage, nutrition-related factors, and low muscle mass. The covariates used in the multivariate analysis were selected from the factors that were significantly different in the univariate analysis.

## Results

### Setting of cutoff values for masseter muscle volume

The MMV cutoff values were calculated as follows: MMV (men, 45.030 cm3/area under the curve [AUC] = 0.690; women, 31.752 cm3/AUC = 0.625). We divided the patients into two groups (low and normal MMV groups/low and normal AIMMV groups) based on these cutoff values.

### Evaluation of manually extracted masseter muscle volume

**Fig. 2:**
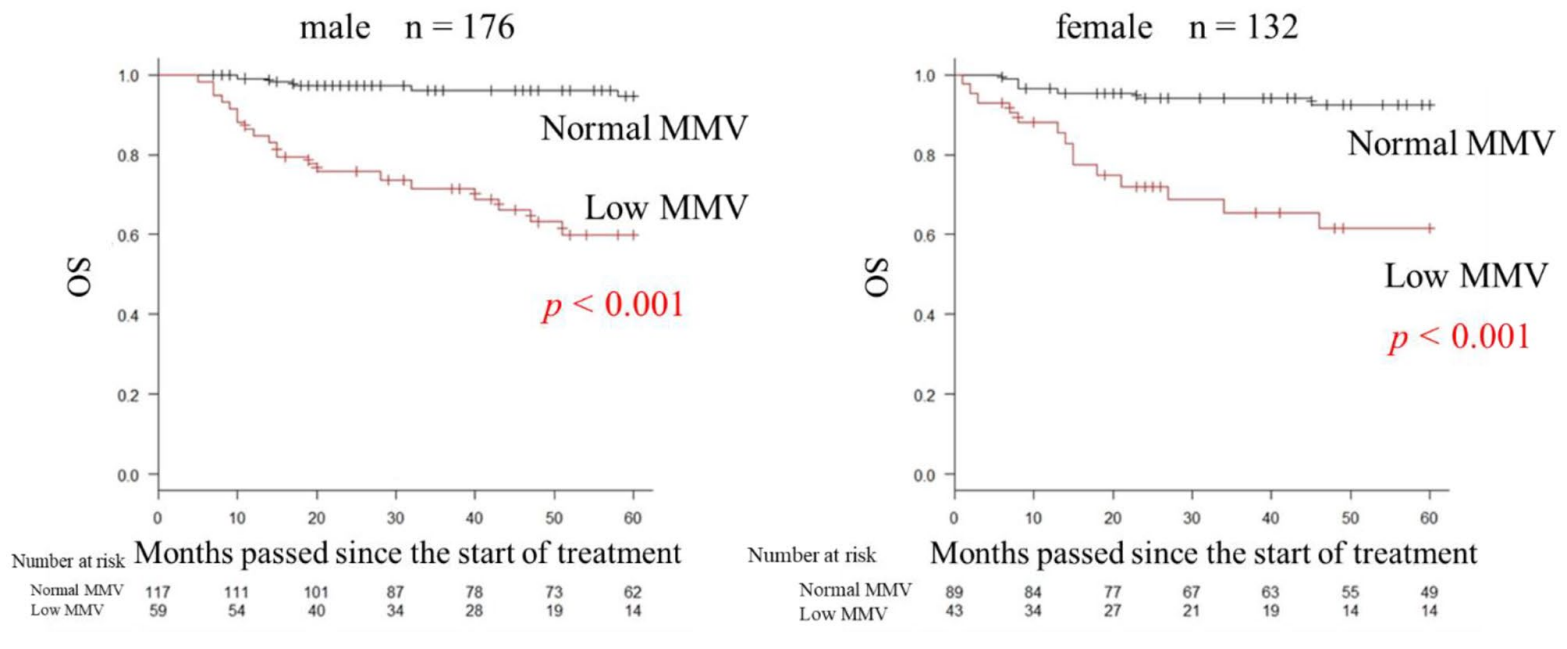
Overall survival by masseter muscle volume (MMV) Both men and women had significantly lower overall survival rate in the low MMV group (men: hazard ratio [HR] = 0.598; 95% confidence interval [CI], 0.438–0.726; *p* < 0.001; women: HR = 0.616; 95% CI, 0.433–0.755; *p* < 0.001)

MMV and SMI showed a moderate positive correlation in both men and women (men: correlation coefficient [r] = 0.37, *p* < 0.001; women: *r* = 0.36, *p* < 0.001). The OS rate in the low MMV group was significantly lower than that in the normal MMV group for both men and women (men: hazard ratio [HR] = 0.598; 95% confidence interval [CI], 0.438–0.726, *p* < 0.001; women: HR = 0.616; 95% CI, 0.433–0.755; *p* < 0.001) (Fig. 2).

In the univariate analysis, stage, CRP, Alb, A/G ratio, mGPS, CONUT score, NLR, PNI, BMI, and low MMV were significantly associated with OS. In the multivariate analysis, low MMV was an independent poor prognostic factor (HR, 4.325; 95% CI, 2.082–8.981; *p* < 0.001) (Table 3).

**Table 3:** Univariate and multivariate analyses in the low MMV group

Variables		Univariate analysis		Multivariate analysis	
		(Fisher’s exact test)		(Cox proportional hazards model)	
	Cutoff	HR	*p*-value	HR	*p*-value
		(95% CI)		(95% CI)	
Age	77	2.018	0.068		
		(0.919–4.268)			
Sex	Male or female	0.927	0.871		
		(0.468–1.857)			
Stage	I and II or III and IV	10.875	< 0.001	5.631	< 0.001
		(4.714–28.304)		(2.477–12.800)	
CRP (mg/dL)	1.15	3.259	0.021	0.659	0.501
		(1.044–9.314)		(0.196–2.220)	
Alb (g/dL)	4.3	2.090	0.035	0.582	0.183
		(1.041–4.329)		(0.262–1.292)	
A/G ratio	1.58	3.015	0.004	1.249	0.617
		(1.315–7.802)		(0.522–2.987)	
T-cho (mg/dL)	143	1.504	0.510		
		(0.349–4.984)			
mGPS	2	12.550	0.005	2.516	0.238
		(1.736–143.109)		(0.543–11.650)	
CONUT	2	4.682	< 0.001	1.872	0.094
		(2.143–10.115)		(0.898–3.904)	
CAR	0.033	2.703	0.065		
		(0.920–10.839)			
NLR	2.398	2.676	0.003	1.028	0.939
		(1.337–5.496)		(0.502–2.108)	
PLR	189.51	1.493	0.263		
		(0.688–3.114)			
PNI	53.888	3.428	0.005	1.857	0.273
		(1.371–10.281)		(0.615–5.609)	
BMI (kg/m^2^)	20.233	3.375	< 0.001	2.055	0.022
		(1.680–6.959)		(1.108–3.812)	
MMV (cm^3^)	Male, 45.030		< 0.001		< 0.001
		8.789		4.325	
	Female, 31.752	(4.078–20.350)		(2.082–8.981)	

Low MMV was an independent poor prognostic factor, along with stage and BMI (HR, 4.325; 95% CI, 2.082–8.981; *p* < 0.001)

### Evaluation of the masseter muscle volume automatically extracted by the masseter muscle volume learning model

**Fig. 3:**
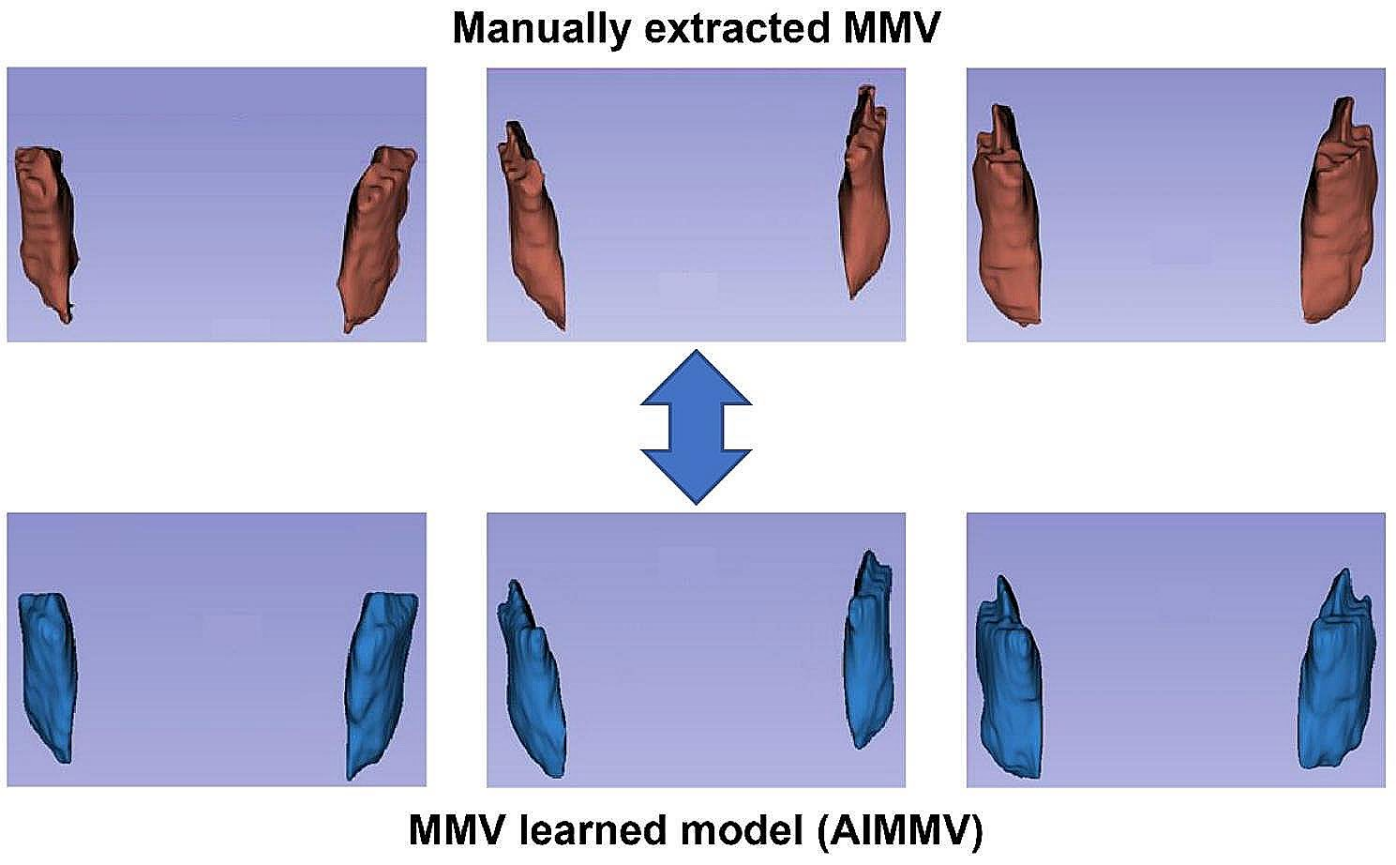
Comparison between masseter muscle volume (MMV) and artificial intelligence MMV (AIMMV)

A comparison of the MMV extracted manually and the AIMMV extracted automatically by the MMV learning model is shown in Fig. 3.

**Fig. 4:**
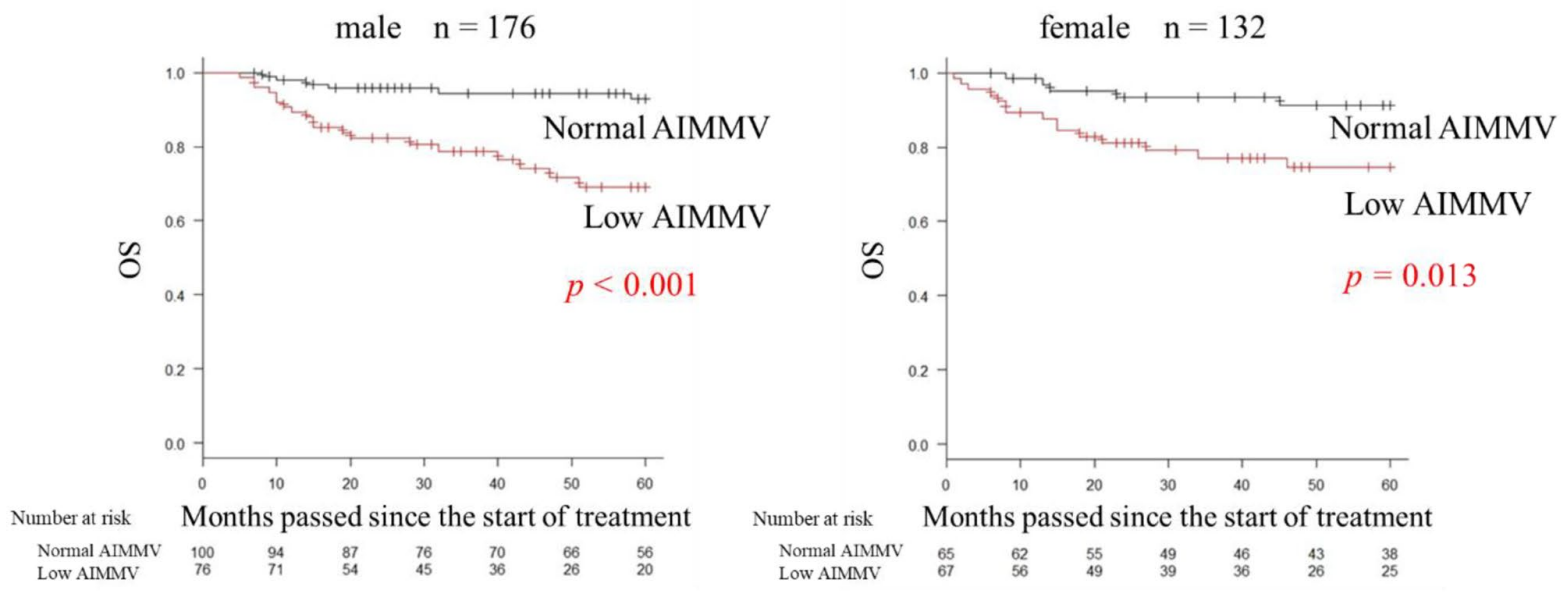
Overall survival based on the artificial intelligence masseter muscle volume In the low AIMMV group, both men and women had significantly lower overall survival rate (men: HR = 0.690; 95% CI, 0.547–0.795; *p* < 0.001; women: HR = 0.746; 95% CI, 0.611–0.840; *p* = 0.013)

MMV and AIMMV showed a high positive correlation in both men and women (men: *r* = 0.972, *p* < 0.001; women: *r* = 0.965, *p* < 0.001). The OS rate in the low AIMMV group was significantly lower than that in the normal AIMMV group for both men and women (men: HR = 0.690; 95% CI, 0.547–0.795; *p* < 0.001; women: HR = 0.746; 95% CI, 0.611–0.840; *p* = 0.013) (Fig. 4).

In the univariate analysis, stage, CRP, Alb, A/G ratio, mGPS, CONUT score, NLR, PNI, BMI, and low AIMMV were significantly associated with OS. In the multivariate analysis, low AIMMV was an independent poor prognostic factor (HR, 2.231; 95% CI, 1.055–4.719; *p* = 0.036) (Table 4).

**Table 4:** Univariate and multivariate analyses of the low AIMMV group

Variables		Univariate analysis		Multivariate analysis	
		(Fisher’s exact test)		(Cox proportional hazards model)	
	Cutoff	HR	*p*-value	HR	*p*-value
		(95% CI)		(95% CI)	
Age	77	2.018	0.068		
		(0.919–4.268)			
Sex	Male or female	0.927	0.871		
		(0.468–1.857)			
Stage	I and II or III and IV	10.875	< 0.001	6.267	< 0.001
		(4.714–28.304)		(2.785–14.100)	
CRP (mg/dL)	1.15	3.259	0.021	0.733	0.615
		(1.044–9.314)		(0.219–2.454)	
Alb (g/dL)	4.3	2.090	0.035	0.704	0.374
		(1.041–4.329)		(0.325–1.525)	
A/G ratio	1.58	3.015	0.004	1.258	0.621
		(1.315–7.802)		(0.507–3.122)	
T-cho (mg/dL)	143	1.504	0.510		
		(0.349–4.984)			
mGPS	2	12.55	0.005	2.942	0.169
		(1.736–143.109)		(0.632–13.710)	
CONUT	2	4.682	< 0.001	2.093	0.040
		(2.143–10.115)		(1.035–4.229)	
CAR	0.033	2.703	0.065		
		(0.920–10.839)			
NLR	2.398	2.676	0.003	0.995	0.988
		(1.337–5.496)		(0.485–2.039)	
PLR	189.507	1.493	0.263		
		(0.688–3.114)			
PNI	53.888	3.428	0.005	1.619	0.405
		(1.371–10.281)		(0.521–5.028)	
BMI (kg/m^2^)	20.233	3.375	< 0.001	2.044	0.023
		(1.680–6.959)		(1.102–3.793)	
AIMMV (cm^3^)	Male, 45.030		< 0.001		0.036
		4.346		2.231	
	Female, 31.752	(2.042–9.946)		(1.055–4.719)	

Low AIMMV was an independent poor prognostic factor, along with stage, CONUT score, and BMI (HR, 2.231; 95% CI, 1.055–4.719; *p* = 0.036)

## Discussion

In recent years, nutritional disorders and sarcopenia have been associated with postoperative complications and life expectancy in patients with various cancers. Several methods for assessing sarcopenia have been reported using the L3-level psoas muscle cross-sectional area on abdominal CT images in patients with gastrointestinal cancer [24–26]. However, abdominal CT is not routinely performed in patients with oral cancer. Swartz et al. [27] reported a sarcopenia assessment method using sternocleidomastoid and paravertebral muscle cross-sectional areas at the level of the third cervical vertebra on head and neck CT images. In 2017, Wallace et al. [9] reported a sarcopenia assessment method using the masseter muscle cross-sectional area on head CT images. Owing to the advancements in diagnostic imaging, volume, rather than cross-sectional area, has be-come an important parameter in assessing sarcopenia in various clinical settings [28, 29]. This study showed a correlation between SMI as defined in the AWGS [6] diagnostic criteria and MMV on CT images. Furthermore, a decrease in MMV on CT images was an independent poor prognostic factor in patients with oral cancer. This study is the first to report a significant association between MMV measured on head and neck CT images and life expectancy of patients with oral cancer. The cross-sectional area and density of the masseter muscle decrease with aging, and these changes are consistent with the general age-related changes in muscle tissue throughout the body [30, 31]. The masseter muscle thickness measured by ultrasound may be related to the risk of malnutrition in older adult patients requiring care [10]. Masseter muscle thickness measured by ultrasound in elderly patients with hip fractures may also be associated with the risk of dysphagia [32]. Masseter muscle atrophy occurs with aging through the activation of the autophagy–lysosome pathway [33]. Hwang et al. demonstrated a significant correlation between the mass of the masseter muscle and that of the L3 psoas major. This finding implies that the masseter muscle mass could be indicative of general muscular mass and nutritional status, considering the pivotal role of the L3 psoas major in the evaluation of sarcopenia [34]. Additionally, various preoperative nutritional indicators in patients with advanced oral cancer have been linked to both the occurrence of Surgical Site Infections and life expectancy [35]. These observations suggest that the Masseter Muscle Volume (MMV) might serve as a valuable prognostic tool in oral cancer cases. On the other hand, it has been reported that patients with oral cancer often experience a decline in oral function and nutritional status due to the effects of the cancer before treatment [36]. Clinically, it is anticipated that the more advanced the cancer stage, the more pronounced these effects become. Therefore, particularly in cases of advanced lower gingival carcinoma, direct invasion of the masseter muscle may be affected, suggesting that our findings may not be applicable in such situations.

These studies suggested that the sarcopenia assessment method using MMV measured on the head and neck CT images may be useful for predicting the life expectancy of patients with oral cancer.

Currently, the extraction of MMV from CT images is performed by manual manipulation, which is cumbersome and may lead to bias. To address this issue, we developed a learning model for the automatic extraction of the MMV using deep learning. The Clara Train SDK used in this study included NVIDIA’s pretrained models (Medical Model Archive [MMAR]). New models can be developed with high accuracy using MMAR for transfer learning. In this study, a high correlation was observed between the MMV in the manual and automatic extraction groups. Furthermore, a decrease in MMV, automatically extracted from the MMV learning model, was an independent poor prognostic factor. Therefore, it is possible to quickly, simply, and objectively predict the prognosis of patients with oral cancer. However, the MMV values automatically extracted by the MMV learning model tended to be relatively lower than those of the manual extraction group. To improve its accuracy, the number of training data should be increased, and the training parameters should be standardized.

This study has some limitations. It is a single-center retrospective study. Due to the relatively small number of patients and unequal proportion of men and women, selection bias cannot be excluded. In addition, the definition of sarcopenia based on muscle mass measured on CT images has not been established, and the specific cutoff values have not been determined [37]. In this study, the target patients were Japanese, and the cutoff values were set using the AWGS [6] diagnostic criteria based on the Asian epidemiological data. Therefore, large prospective studies are required to validate the usefulness of MMV in patients with oral cancer. As this study focused only on patients with oral cancer, further studies will be conducted to validate and analyze the association of various factors in other patient groups.

## Conclusions

This study showed that assessing sarcopenia using MMV measured on CT images is associated with life expectancy in patients with oral cancer. Furthermore, the method for assessing sarcopenia using the MMV learning model developed utilizing deep learning has also been associated with life expectancy. Therefore, this study suggests that the sarcopenia assessment method using MMV measured on CT images and the MMV learning model may be useful for predicting the life expectancy in patients with oral cancer.

## Data Availability

The datasets generated and/or analyzed during the current study are not publicly available due to ethical concerns regarding patient’s confidentiality but are available from the corresponding author on reasonable request.

## Abbreviations

A/G: Alb/globulin
AI: Artificial intelligence
AIMMV: Artificial intelligence masseter muscle volume
Alb: Albumin
AWGS: Asian Working Group for Sarcopenia
BIA: Bioelectrical impedance analysis
BMI: Body mass index
CAR: CRP/Alb ratio [16]
C3: Cervical
Clara: Clara Train SDK from NVIDIA Clara Imaging
CONUT: And controlling nutritional status
CRP: C-reactive protein
CT: Computed tomography
HR: Hazard ratio
L3: Third lumbar vertebra
mGPS: Modified Glasgow prognostic score
MMAR: Medical Model Archive
MMV: Masseter muscle volume
NLR: Neutrophil/lymphocyte ratio
OS: Overall survival
PLR: Plate-let/lymphocyte ratio
PNI: Prognostic nutrition index
ROC: Receiver operating characteristic
SD: Standard deviation
SMI: Skeletal muscle index
SMI: Skeletal muscle index
T-cho: Total cholesterol
